# 

**Published:** 1894-08-04

**Authors:** 


**Notices of Births, Deaths, and Marriages** are inserted in this column at a charge of 2s. 6d. for an announcement not exceeding four lines.

**Notice to Advertisers and Subscribers.**

All Advertisements, Orders for Copies of The Hospital, and Business Communications should be addressed to **The Publisher, NOT TO THE EDITOR,** The Hospital, 428, Strand, W.C.

The Cost of Subscription, post free, is as follows:—Per week, 2½d.; per quarter, 2s. 8d.; per half-year, 5s. 3d.; per annum, 10s. 6d. Foreign:—Per Annum, 12s. 8d.; payable, in all cases, in advance.

### NOTICE TO CORRESPONDENTS.

All MS., Letters, Books for Review, and other matters intended for the Editor should be addressed The Editor, The Lodge, Porchester Square, London, W.

The Editor cannot undertake to return rejected MS., even when accompanied by stamped directed envelope.

**"Burdett's Hospital Annual," 1894.**

Fifth year of publication. 900 pp. Boards, gilt lettering. A most complete guide to British, Colonial, Indian, and American Hospitals, Dispensaries, Nursing and Convalescent Institutions, and Asylums. Price 5s. Published by The Scientific Press (Limited), 428, Strand, W.C.

**NOTICE.—The Index for Vol. XV. (ending March 31st, 1894) is on sale at the Office of this Journal, price 2½d., post free.**

### SCRAPS AND GLEANINGS.

The sixty-second annual meeting of the British Medical Association was opened at Bristol on the 31st of last month.

The New British Home for Incurables at Streatham has received £1.000 from a gentleman desirous of endowing a bed.

The Commissioners in Lunacy report an increase in lunacy during the past year of 2,245. In the county of London alone the increase amounts to 800.

The Bishop of London has appealed on behalf of the Children's Country Holiday Fund. It is shown that £5 will make ten children happy for a fortnight.

A new municipal lodging-house has been erected at Croydon at a cost of about £6,500. The cubical system has been adopted, and all the sanitary arrangements are excellent.

The Secretary of the South London Association for Assisting the Blind is appealing for subscriptions to enable the association to give their blind members their annual summer outing.

The friendly societies at Ripon united on Hospital Sunday in an amalgamated demonstration, and divine service was attended in the Cathedral. The collections amounted to over £26.

The Horton Infirmary benefited to the extent of £30 from the Hospital Saturday collections at Banbury. The Horton Infirmary commenced twenty years ago with twelve beds, and now there are thirty.

In connection with the opening of the New British Home for Incurables at Streatham by the Princess of Wales on the 3rd ult., a gentleman forwarded a donation of £1,000 to endow a bed "in memory of his brother."

The Board of Management of the British Home for Incurables have received a donation of £100 from the Worshipful Company of Grocers, and £5 5s. from the Worshipful Company of Joiners' to assist in opening the new home free of debt.

As a result of recent revelations in the French press concerning the insanitary conditions of French deep-sea fishermen, strong efforts are being made to obtain and equip for them a series of hospital boats provided with doctors and all medical appliances.

The Swindon Victoria Hospital is about to effect a considerable extension of their accommodation. A larger operating room is to be constructed and two new wards provided. The contract for the whole work is £700, of which the committee have £500 in hand.

The Lord Mayor is receiving subscriptions for the relief fund of the sufferers from the earthquake in Constantinople. A donation of £500 has been sent by Messrs. N. M. Rothschild and Sons, and the same sum from Messrs. de Rothschild Brothers in Paris.

At the quarterly meeting of the governors of the Leicester Infirmary the representative of the Sports Committee said that they were in a position this year to hand over £300 for the benefit of the infirmary. The institution has received altogether £4,000 from the Sports Committee.

A garden fête was lately held in the grounds of Buccleuch House, Richmond, to swell the funds of the Royal Cambridge Asylum for Soldiers' Widows. The estimated annual expenditure amounts to £3,000, the funded income being £800, with an additional £50 from the Princess Mary's Fund for Nurses.

At the annual meeting of the governors of the Birmingham and Midland Eye Hospital it was stated that the land for the extension of the hospital had been taken and arrangements made for proceeding with the work at an early date. The statement of accounts was most favourable, the balance in hand being £6,065 12s. 10d.

Special chambers for medical men are being constructed in New York. No other occupants are to be admitted. The building is situated in a central part of New York, and is to be well provided with telephonic communication. The "entresol" is to be occupied by chemists, surgical instrument shops, and a nursing agency for the town.

The Essex County Council have recently held a meeting to consider the question of isolation hospitals. It seems that each parish wants permission to put up its isolation hospital in another parish. The Council decided to reappoint an officer of the county to tabulate the returns of the local medical officers of health, and to report to them at the next meeting.

At the ordinary meeting of the managers of the Metropolitan Asylums Board, plans for the proposed alterations and additions for the Westminster Hospital were passed, and a letter was read from the Local Government Board authorising the managers to expend the sum of £13,150 to increase the accommodation for patients and staff at the Eastern Hospital.

In view of the spreading of fever contagion by infected tramps, it was suggested at the meeting of medical officers and sanitary inspectors "that power should be given to the local authorities to require medical examinations of all persons entering common lodging-houses and casual wards, and that each inmate on entering be required to have a bath of fresh water."

The special course of lectures instituted by the National Health Society will recommence in October next at 53, Berners Street, W. The training is intended to qualify women to compete for lectureships on nursing and health under the County Council scheme. The lectures will include the subjects of anatomy, physiology, sanitation, hygiene, first aid to the injured, and nursing.

At the annual meeting in connection with the Northumberland, Durham, and Newcastle Eye Infirmary the financial condition was shown to be very unsatisfactory, the debit of the infirmary's account with the treasurer amounting to £263 11s. 2d. It is hoped that the public will come forward and remedy a condition of accounts which may necessitate the closing of several beds.

Steps are being taken by the leading physicians and philanthropists of New York to provide a hospital for incurable consumptives. The City Board of Health are taking measures for the inspection and disinfection of premises previously or now occupied by consumptive persons, and 15,000 copies of "Instruction to Consumptives" are to be printed and distributed in English, German, Italian, and Hebrew.

### THE STUDENT OF MEDICINE.

### APPOINTMENTS.

R. Aldous, L.R.C.P., L.R.C.S.Edin., L.F.P.S.Glasg., appointed Honorary Surgeon to the Beccles Hospital. D. C. Bremner, M.B., C.M.Edin., appointed Assistant Medical Officer to the East Riding Lunatic Asylum, Beverley. E. Gardner, L.D.S., appointed Dental Surgeon to Westminster Hospital. W. R. Grove, M.D.St.And., M.R.C.S.Eng., reappointed Medical Officer of Health for the St. Ives Urban Sanitary District. H. L. Hatch, M.R.C.S., L.R.C.P.Lond., D.P.H., appointed House Surgeon to the Fleming Memorial Hospital for Children, Newcastle-on-Tyne. H. P. Helsham, M.R.C.S., L.R.C.P.Lond., appointed Honorary Surgeon to the Beccles Hospital. Dr. G. F. Johnston, appointed Physician to the Out-patients at the Hospital for Epilepsy and Paralysis, Regent's Park. A. E. Joscelyne, L.R.C.P., L.S.A., appointed Surgeon to the South Metropolitan Gas Company, West Greenwich Works. J. Keay, M.D.Glasg., appointed Medical Superintendent to the Northern Counties District Asylum, Inverness. Mr. R. L. Knaggs, M.A., M.D., M.C.Cantab., F.R.C.S., appointed an Honorary Assistant Surgeon to the General Infirmary, Leeds. J. T. Knight, M.R.C.S.Eng., reappointed Medical Officer of Health to the Carlton Local Board. C. D. Marshall, F.R.C.S., appointed Pathologist to the Royal London Ophthalmic Hospital, Moorfields. W. N. Puddicombe, M.R.C.S.Eng., appointed Honorary Medical Officer to the St. Albans Hospital. A. J. Tonkin, M.B., B.Ch.R.U.I., appointed Resident Medical Officer to the Clayton Vale Small-pox Hospital, Clayton Vale, Manchester. P. M. Yearsley, F.R.C.S., appointed Lecturer on Surgical Pathology to Westminster Hospital Medical School.

### VACANCIES.

#### Resident Medical Officers.

Central London Ophthalmic Hospital, 238a, Gray's Inn Road, W.C.—House Surgeon. Rooms, coals, and light provided. Applications to the Secretary by August 7th.

Bath Eastern Dispensary.—Resident Medical Practitioner. Salary £100 per annum, with furnished apartments, coal, gas and domestic attendance. Applications to Colonel F. V. Eyre, R.A., Honorary Secretary, Rockville, Lansdown, Bath, by August 11th.

Chichester Infirmary.—House Surgeon. Salary £80 per annum, with board, lodging, and washing. Applications to Eugene Street, Secretary, by August 6th.

General Hospital, Nottingham.—Senior Resident Medical Officer; doubly qualified. Salary £120 for the first year, with an addition of £10 a year up to £150, with board, residence, and washing. Applications to the Secretary by September 1st.

North London Consumption Hospital, Hampstead, N.W.—Resident Medical Officer; doubly qualified. Honorarium £40 per annum, with board, rooms, &c., in the hospital. Applications to Lionel F. Hill, Secretary, at the office, 41, Fitzroy Square, W., by August 4th.

Sussex County Hospital, Brighton.—House Surgeon; doubly qualified, unmarried, and under 30 years of age. Salary £120, rising to £140 per annum, with board, residence, and washing. Applications to the Secretary by August 7th.

Sussex County Hospital, Brighton.—Assistant House Surgeon, unmarried, and under 30 years of age. Emoluments are a salary not exceeding £30 per annum, with board, washing, and residence. Applications to the Secretary by August 15th.

Western General Dispensary, Marylebone Road, N.W.—Junior House Surgeon, unmarried. Salary £50 per annum, with board and lodging. Applications to the Honorary Secretary by August 6th.

West Riding Asylum, Wakefield.—Fourth Assistant Medical Officer. Salary £100 per annum, rising £10 annually to a maximum of £150, with furnished apartments, board, washing, and attendance. Applications to the Medical Director by August 18th.

#### Non-Resident Medical Officers.

Cambridge University.—John Lucas Walker Studentship. Applications to Professor Roy, New Museum, Cambridge, by September 30th.

Chelsea Hospital for Women, Fulham Road, S.W.—Physicians to In-patients, Physicians to Out-patients, and Pathologist. Forms of application, to be returned to the Secretary, A. C. Davis, by August 6th.

Rubery Hill Asylum, Bromsgrove, Worcestershire.—Clinical Assistant. Applications to Medical Superintendent.

### THE STUDENT OF MEDICINE—*continued.*

### Victoria University.

"Degree Day."—"Degree Day" in connection with Victoria University was held on June 30th. The candidates who had qualified for degrees were :—

Doctor of Science.—J. Beard, Owens.

Doctors of Medicine.—R. Alcock, J. C. Buckley, and J. H. Crocker, Owens.

Masters of Science.—Owens College: W. A. Bone, F. C. Moore, T. E. Stanton, R. G. Wild, and G. Wilson. University College: A. Carey and J. C. Quinn.

Bachelor of Science (Honours).—W. C. Varley, Owens.

Bachelors of Medicine and of Surgery.—Owens College: W. J. Bowden (received the degree in March, and is presented only), J. Jones. University College: E. D. Minshall.

### University of Dublin.

At the summer commencement (Comitia Æstiva) of Trinity Term, held in the Theatre, or Examination Hall, of Trinity College, on Thursday, June 20th, 1894, the following degrees (amongst others) were conferred by the University Caput in the presence of the Senate :—

Licentiatus in Medicina, in Chirurgia, et in Arte Obstetricia.—M. J. Smith.

Baccalaureus in Medicina.—W. J. Thompson.

Baccalaurei in Medicina, in Chirurgia, et in Arte Obstetricia.—C. Brew, M. C. Carmichael, A. R. Friel, E. E. Goodbody, F.W. Goodbody, R. R. Hastings, H. Kirkpatrick, H. C. Mactier, R. W. Merrick, A.C. O'Sullivan (stip. cond.), A. J. H. Thornton, J. W. Watkins, F. F. Wynne.

Doctor in Scientiis.—D. Morris.

Doctores in Medicina.—H. E. Blandford, J. W. Boyce, H. C. Drury (stip. cond.), W. W. Fenton, R. P. Jones, M. T. Kelly, L. G. F. Macrory, W. A. Valentine, C. E. W. Wilmot.

Honorarium £40 per annum, with Board, Rooms, &c., in the Hospital.

CHELSEA HOSPITAL FOR WOMEN,

BATH—EASTERN DISPENSARY.

